# Combination of umbilical cord mesenchymal stem cells and standard immunosuppressive regimen for pediatric patients with severe aplastic anemia

**DOI:** 10.1186/s12887-021-02562-x

**Published:** 2021-02-27

**Authors:** Yang Lan, Fang Liu, Lixian Chang, Lipeng Liu, Yingchi Zhang, Meihui Yi, Yuli Cai, Jing Feng, Zhibo Han, Zhongchao Han, Xiaofan Zhu

**Affiliations:** 1 State Key Laboratory of Experimental Hematology, National Clinical Research Center for Blood Diseases, Institute of Hematology & Blood Diseases Hospital, Chinese Academy of Medical Sciences & Peking Union Medical College, 288 Nanjing Road, Heping District, Tianjin, 300020 China; 2 National Engineering Research Center of Cell Products, Tianjin AmCellGene Engineering Co., Ltd, Tianjin, 300020 China

**Keywords:** Child, Immunosuppressive therapy, Mesenchymal stem cell, Severe aplastic anemia, Umbilical cord

## Abstract

**Background:**

Defects of bone marrow mesenchymal stem cells (BM-MSCs) in proliferation and differentiation are involved in the pathophysiology of aplastic anemia (AA). Infusion of umbilical cord mesenchymal stem cells (UC-MSCs) may improve the efficacy of immunosuppressive therapy (IST) in childhood severe aplastic anemia (SAA).

**Methods:**

We conducted an investigator-initiated, open-label, and prospective phase IV trial to evaluate the safety and efficacy of combination of allogenic UC-MSCs and standard IST for pediatric patients with newly diagnosed SAA. In mesenchymal stem cells (MSC) group, UC-MSCs were injected intravenously at a dose of 1 × 10^6^/kg per week starting on the 14th day after administration of rabbit antithymocyte globulin (ATG), for a total of 3 weeks. The clinical outcomes and adverse events of patients with UC-MSCs infusion were assessed when compared with a concurrent control group in which patients received standard IST alone.

**Results:**

Nine patients with a median age of 4 years were enrolled as the group with MSC, while the data of another 9 childhood SAA were analysed as the controls. Four (44%) patients in MSC group developed anaphylactic reactions which were associated with rabbit ATG. When compared with the controls, neither the improvement of blood cell counts, nor the change of T-lymphocytes after IST reached statistical significance in MSC group (both *p* > 0.05) and there were one (11%) patient in MSC group and two (22%) patients in the controls achieved partial response (PR) at 90 days after IST. After a median follow-up of 48 months, there was no clone evolution occurring in both groups. The 4-year estimated overall survival (OS) rate in two groups were both 88.9% ± 10.5%, while the 4-year estimated failure-free survival (FFS) rate in MSC group was lower than that in the controls (38.1% ± 17.2% vs. 66.7% ± 15.7%, *p* = 0.153).

**Conclusions:**

Concomitant use of IST and UC-MSCs in SAA children is safe but may not necessarily improve the early response rate and long-term outcomes. This clinical trial was registered at ClinicalTrials.gov, identifier: NCT02218437 (registered October 2013).

## Background

Severe aplastic anemia (SAA) is a potentially fatal disease characterized by hypoplastic or fatty bone marrow (BM), with peripheral blood (PB) pancytopenia [1]. A good deal of evidence have indicated that immune-mediated T cell destruction of hematopoietic stem cells (HSCs) is involved in the pathogenesis of acquired aplastic anemia (aAA) [2, 3]. Furthermore, the number of circulating regulatory T cells (Tregs) is generally decreased in patients with aAA [4–6]. For SAA, hematopoietic stem cell transplantation (HSCT) from a matched sibling donor is still the first-line treatment nowadays, however, due to lack of human leukocyte antigen (HLA)-compatible donors, immunosuppressive therapy (IST) with antithymocyte globulin (ATG) and cyclosporine (CsA) is also widely used in clinical practice for its potential role in young patients [7] and older patients [3]. However, the efficacy of IST is limited due to lack of response, relapse, and clonal evolution [8]. The early response rate of standard IS regimen in childhood SAA (40-50 days after ATG treatment) is correlated with long-term efficacy.

Mesenchymal stem cells (MSCs) are plastic-adherent, characterized by expression of cell surface antigens (CD105, CD73, and CD90), and lack of CD34, CD45, CD14 or CD11b, CD79α or CD19 and HLA-DR surface molecules. As essential components of bone marrow microenvironment, they can support HSCs growth, and with the potential of differentiating in vitro into adipocytes, osteoblasts, and chondroblasts [9–11]. They are capable of modulating the activity of immune cells such as T cells, B cells, natural killer cells, and dendritic cells [12]. The sources of MSCs are various and they can be obtained from various tissues, including BM, dental pulp tissue, adipose tissue, amniotic fluid, and umbilical cord tissue [13–16]. It has been demonstrated that defects of BM-MSCs in proliferation and differentiation play a pivotal role in the pathophysiology of AA [17]. Nowadays, with the deep research on MSCs, the clinical application of MSCs which aims to restore the BM hematopoietic microenvironment, has provided researchers with a new treatment model for AA. Several studies had suggested that treatment with MSCs may be a feasible and effective therapeutic option for AA patients [18, 19]. Nevertheless, the application of MSCs limited to childhood SAA was rare.

Umbilical cord mesenchymal stem cells (UC-MSCs), which are isolated from healthy human umbilical cord tissue, may be ideal source of MSCs due to their abundant supply, painless collection procedures, and great expansion ability. In our previous study, a child with a 4-year history of SAA was administrated with culture-expanded allogenic UC-MSCs after the IST of cyclophosphamide (30 mg/kg/day× 4 days) and CsA (5 mg/kg/day). The patient reaches transfusion independence now and no serious side effect occurs during or after the infusion of UC-MSCs. It has illustrated that infusion of UC-MSCs may improve the efficacy of IS therapy in childhood SAA. To assess the safety and efficacy of UC-MSCs infusion for pediatric patients with SAA, we further conducted an investigator-initiated, open-label, and prospective phase IV clinical trial.

## Methods

### Study population

Patients aged between 1 month to 18 years with newly diagnosed SAA were consecutively recruited from October 2013 to October 2014 at the Institute of Hematology and Blood Diseases Hospital (China). SAA was defined as the BM cellularity < 25% (or 25-50% with < 30% residual haematopoietic cells) and at least two of the following three criteria: absolute neutrophil count (ANC) < 0.5 × 10^9^/L, pre-transfusion platelet (PLT) count < 20 × 10^9^/L, or pre-transfusion absolute reticulocyte count (ARC) < 20 × 10^9^/L. Very severe aplastic anemia (vSAA) was considered adopting the same criteria, with ANC < 0.2 × 10^9^/L [20, 21]. Patients underwent complete medical history and physical examination, routine cytogenetic analysis, mitomycin C testing, comet assay, and next-generation sequencing methods to exclude inherited bone marrow failure syndrome such as Fanconi anemia, Diamond Blackfan anemia and dyskeratosis congenital. Subjects with a HLA-matched sibling donor for immediate HSCT were also excluded. Other eligibility criteria encompassed adequate hepatic and renal functions, and ECOG performance status of ≤2.

The study was approved by the Ethics Committee and Institutional Review Board of Chinese Academy of Medical Sciences and Peking Union Medical College, and conducted in concordance with the Declaration of Helsinki. All legal guardians of pediatric patients signed written informed consent before participation in the trial.

### Study design

This was an investigator-initiated, open-label, single-center and prospective phase IV trial. The study was registered at ClinicalTrials.gov, identifier NCT02218437. Eligible patients were divided into two treatment groups based on their guardians’ choices. Patients in MSC group received standard IST combined with UC-MSCs, while patients as the controls received standard IST alone. Rabbit ATG (Thymoglobuline®, IMTIX-SANGSTAT) (3 mg/kg.d × 5 days) and CsA (5 mg/kg.d) were administered in both groups, and only in MSC group, UC-MSCs were injected intravenously at a dose of 1 × 10^6^/kg per week starting on the 14th day after rabbit ATG, for a total of 3 weeks. UC-MSCs in this study were supplied free of charge by Tianjin AmCellGene Engineering Co., Ltd. The primary objective of this work was to investigate the tolerability and toxicities of UC-MSCs for pediatric patients with SAA. The secondary objective was to evaluate the efficacy of UC-MSCs plus standard IST in childhood SAA.

### Clinical evaluation and response criteria

Participants underwent baseline evaluations that comprised a complete medical history and physical examination, blood counts and serum biochemistry; BM aspiration and biopsy; serum immunoglobulins level; percentage of T-cell subsets in PB; detection of paroxysmal nocturnal hemoglobinuria (PNH) clone by flow cytometry in PB; cytogenetic and mutational analysis; and next-generation sequencing methods. Blood counts and serum biochemistry were monitored and BM aspiration and/or biopsy for morphology and cytogenetics was also performed at 40 days, 3 months and 6 months after rabbit ATG.

Hematologic response was defined as follows: complete response (CR) was defined as ANC > 1.5 × 10^9^/L, PLT > 100 × 10^9^/L, and hemoglobin (HGB) normal for age (all three criteria had to be achieved); partial response (PR) was defined as transfusion independence, ANC > 0.5 × 10^9^/L, PLT > 20 × 10^9^/L, and ARC > 30 × 10^9^/L above the baseline; and persistence of transfusion dependence or death was evidence of nonresponse (NR) [22]. Failure-free survival (FFS) was defined as the time from initiation of rabbit ATG until the date of treatment failure, relapse or death, whichever came first.

### Measurement of Tregs

CD4 + CD25 + CD127dim Tregs were identified by flow cytometric analysis. CD4-peridinin chlorophyll (PerCP)-Cy5.5, CD25-phycoerythrin (PE), CD127-allophycocyanin (APC), mouse IgG1-PE, and mouse IgG1-APC antibodies (BD Biosciences, Franklin Lakes, NJ, USA) were used for surface staining. Fresh ethylenediaminetetraacetic acid (EDTA) anticoagulant blood samples (100 μL) were separated into 2 TruCount tubes (BD Biosciences). One tube was stained with 5 μL of CD4-PerCP-Cy5.5, CD25-PE, CD127-APC antibodies, while another was stained with 5 μL of CD4-PerCP-Cy5.5, mouse IgG1- PE, and mouse IgG1- APC as a negative control. After incubation in the dark for 15 min at room temperature, cells were incubated with 450 μL Optilyse C Lysing Solution (BD Biosciences) for 10 min at room temperature and washed three times with phosphate buffered saline (PBS). Data acquisitions were performed on a FACSCantoII flow cytometer (BD Biosciences) and analyzed by FlowJo software (TreeStar, Ashland, OR, USA).

### Statistical analysis

The statistical analysis was performed utilizing IBM SPSS statistics, version 18.0.0.1 (SPSS, Chicago, IL, USA). Continuous variables were presented as the means ± standard deviations or median (range), and categorical variables were expressed as the frequency (percentage). The Kaplan-Meier method was adopted to estimate the FFS rate. Student’s t-test, Chi-square test and Log-rank test were applied to calculate the differences of subgroups. All statistical tests with *p* values < 0.05 were considered significant. Adverse events were graded and reported according to the CTEP Version 4.0 of the NCI Common Terminology Criteria for Adverse Events.

## Results

### Patient characteristics

A total of 18 participants were included in this study according to the eligibility criteria, and was divided into two subgroups. A total of nine patients with newly diagnosed SAA were regarded as the MSC group. The median age at diagnosis was 4 years (range, 1-10). The ratio of males to females was 3:6, and 7 (78%) patients were diagnosed as vSAA. The median duration from diagnosis to initiation of rabbit ATG was 26 days (range, 9-159). At baseline, the median values for ANC, PLT, HGB, and ARC were 0.07 × 10^9^/L (range, 0-0.36), 10 × 10^9^/L (range, 1-18), 76 g/L (range, 47-117), and 5.4 × 10^9^/L (range, 2.6-32.8) respectively. All patients in MSC group completed three doses of allogenic UC-MSCs infusion.

Furthermore, we additionally performed an analysis of 9 pediatrics with SAA who received standard IST alone as the controls. Basically, there were well-matched demographic and clinical features at baseline between two groups (Table 1).

**Table 1:** Baseline characteristics of patients with SAA treated with or without MSCs

**Characteristics**	**MSC Group (*n* = 9)**	**Control Group (*n* = 9)**	***P***
Median age, years (range)	4 (1-10)	5 (3-9)	0.438
Gender, male / female	3 / 6	5 / 4	0.637
Type of AA			
SAA, n (%)	2 (22%)	4 (44%)	0.620
vSAA, n (%)	7 (78%)	5 (56%)	
Median duration from diagnosis to ATG treatment, days (range)	26 (9-159)	39 (26-179)	0.281
Median WBC, ×10^9^/L (range)	1.84 (0.57-4.79)	2.60 (0.65-5.23)	0.699
Median HGB, g/L (range)	76 (47-117)	65 (47-105)	0.475
Median PLT,×10^9^/L (range)	10 (1-18)	8 (4-19)	0.500
Median ANC,×10^9^/L (range)	0.07 (0-0.36)	0.13 (0.01-0.41)	0.229
Median ARC,×10^9^/L (range)	5.4 (2.6-32.8)	4.5 (2.2-17.0)	0.587
Median percentage of CD3 + CD4+ T-cell lymphocytes, % (range)	39.6 (35.7-42.9)	43.2 (28.4-73.0)	0.401
Median percentage of CD3 + CD8+ T-cell lymphocytes, % (range)	25.6 (14.1-35.1)	25.8 (9.4-35.2)	0.885
Median percentage of Tregs, % (range)^a^	7.0 (3.5-9.4)	6.7 (2.2-16.8)	0.763
Median percentage of mature lymphocytes in BM, % (range)	94.1 (44.5-98.5)	87.9 (36.2-97.7)	0.306

*Abbreviations*: *AA* Aplastic anemia, *SAA* Severe aplastic anemia, *vSAA* Very severe aplastic anemia, *ATG* Antithymocyte globulin, *WBC* White blood cell, *HGB* Hemoglobin, *PLT* Platelet, *ANC* Absolute neutrophil count, *ARC* Absolute reticulocyte count, *Tregs* CD4 + CD25 + CD127dim regulatory T cells, *BM* Bone marrow

^a^The normal range of Tregs is 5-10%

### Safety and tolerability

All patients were available for toxicity assessment (Table 2). In general, two groups had similar incidence of side effects. When compared with the controls, the most obvious adverse event in MSC group was anaphylaxis (including drug fever). A total of 4 (44%) patients in MSC group suffered anaphylactic reactions (three with grade 1-2 and one with grade 3), and one of them complicated with grade 2 oral mucositis. However, the anaphylactic reactions (including drug fever) were associated with rabbit ATG, which were mild and can be ameliorated with antiallergic agent. A patient with oral mucositis was with a rather low ANC after UC-MSCs infusion, which may make her more susceptible to infection. In both groups, serious infectious complications (grade ≥ 3) were uncommon, which were happening in 1/9(11%) patients. No other severe side effects were reported during the follow-up period.

**Table 2:** Most common adverse events irrespective of attribution

Adverse Event	**MSC Group (*n* = 9)**		**Control Group (*n* = 9)**	
	Grade 1/2 No. (%)	Grade 3/4 No. (%)	Grade 1/2 No. (%)	Grade 3/4 No. (%)
Hepatotoxicity	0	0	0	0
Renal failure	0	0	0	0
Nausea	0	0	0	0
Diarrhea	0	0	0	0
Oral mucositis	1 (11)	0	0	0
Arrythmia	0	0	0	0
Heart failure	0	0	0	0
Hypertension	0	0	0	0
Pericarditis	0	0	0	0
Peripheral neuropathy	0	0	0	0
Infections	0	1 (11)	0	1 (11)
Anaphylaxis (including drug fever)	3 (33)	1 (11)	0	0
Local phlebitis	0	0	0	0
Fatigue	0	0	0	0
Headache	0	0	0	0
Fingernail changes	0	0	0	0

### Hematologic and immunologic response

**Fig. 1:**
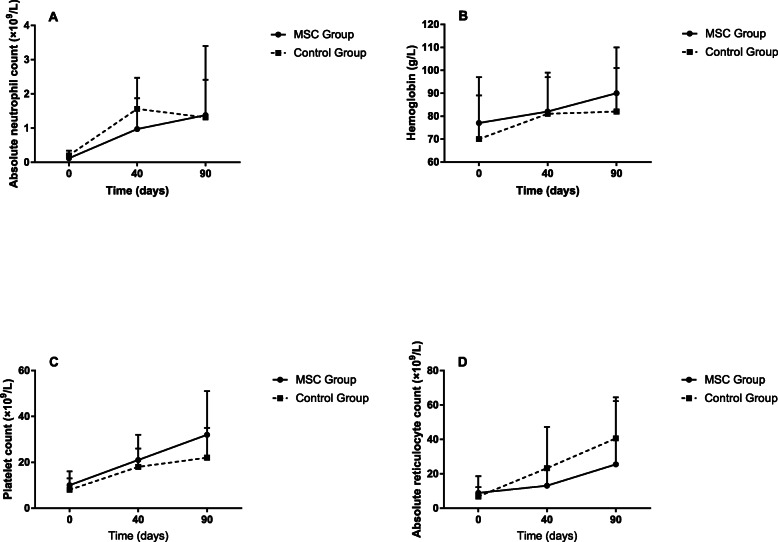
Peripheral blood cells were quantified at baseline, at 40 days and 90 days after rabbit ATG in two treatment groups. **a**-**d** show the changes of absolute neutrophil count, hemoglobin, platelet count and absolute reticulocyte count, respectively

Only 1 (11%) patient in MSC group and 2 (22%) patients in control group achieved PR after 90 days of rabbit ATG. Figure 1 showed the blood cell counts of two groups before treatment, at 40 days and 90 days after rabbit ATG. The increase of PLT levels in MSC group was higher than that in the controls. However, two-factor repeated-measures analysis of variance revealed that improvement of PLT between two groups was of no statistical significance (*p* = 0.192). Meanwhile, there was no significant difference in escalation of ANC (*p* = 0.532), HGB (*p* = 0.450) or ARC (*p* = 0.313) between two groups.

**Fig. 2:**
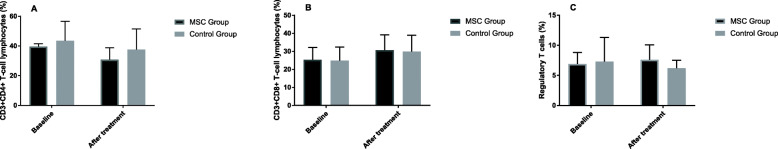
The percentage of CD3 + CD4+ T-cell lymphocytes (**a**), CD3 + CD8+ T-cell lymphocytes (**b**), and CD4 + CD25 + CD127dim regulatory T cells (**c**) in the peripheral blood of patients performed by means of flow cytometry at baseline and after treatment

After 40 days of the initiation of rabbit ATG, the percentage of CD3 + CD4+ T-cell lymphocytes in both groups and CD4 + CD25 + CD127dim Tregs in the controls tended to decrease, while the percentage of CD3 + CD8+ T-cell lymphocytes in both groups and Tregs in MSC group tended to increase (Fig. 2). However, there was no statistically significant change in the percentage of CD3 + CD4+ (*p* = 0.190), CD3 + CD8+ (*p* = 0.875) or CD4 + CD25 + CD127dim (*p* = 0.165) lymphocytes after treatment between two groups.

### Long-term outcomes

**Fig. 3:**
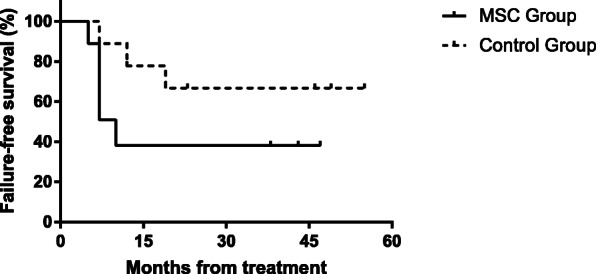
The estimated failure-free survival (FFS) curves of patients between two groups

For all patients, the follow-up endpoint was April 13th, 2018. The median follow-up was 48 months (range, 5-54). One patient in MSC group died of serious infection after 5 months of treatment, while one patient in control group died of intracranial hemorrhages after 7 months of treatment. Both of them were vSAA patients. Three responders (1 in MSC group and 2 in control group) who achieved PR after 90 days of ATG were all alive at follow-up without transplantation. Treatment failure was found in 4 (44%) patients in MSC group and 2 (22%) patients in control group. All of these patients received HSCT as salvage treatment due to lack of response to IST. Among these patients, five children received HSCT from haploidentical family donors and one from a matched unrelated donor. In MSC group, four patients suffered treatment failure and underwent HSCT between 7 and 10 months after IST. One of them experienced grade 3 acute graft-versus-host disease (aGVHD) in intestinal, grade 2 aGVHD in skin and eye (conjunctival hyperemia with chemosis, and increased mucus secretion), and chronic graft-versus-host disease (cGVHD) in joint (joint stiffness and restricted range of motion in knee) after HSCT. At the follow-up endpoint, the patient still suffered from cGVHD in eye and was treated with oral tacrolimus and methylprednisolone. The other five patients who received HSCT were all alive without cGVHD at last follow-up. There was no clone evolution or relapse occurring in both groups at the follow-up endpoint. The 4-year estimated overall survival (OS) rate in two groups were both 88.9 ± 10.5%. The 4-year estimated FFS rate in MSC group was lower than that in the controls (38.1 ± 17.2% vs. 66.7 ± 15.7%). However, there was no significant difference between two groups (*p* = 0.153) (Fig. 3).

## Discussion

SAA is a rare but heterogeneous disorder. Recent studies have illustrated that haematopoietic stem/progenitor cells (HSPCs) and BM-MSCs from patients with aAA possess intrinsic deficits that contribute to their vulnerability in disease evolution [23]. Several studies have revealed the feasibility and efficacy of MSCs in improving matrix microenvironment and promoting hematopoietic recovery in AA patients who underwent HSCT [24–26]. Nevertheless, in SAA patients who lack a histocompatible sibling donor, the mainstay of treatment is still the IST. There were only few published works about combining MSCs infusion with IST for SAA treatment [18, 19, 27]. Our study is the first clinical trial to assess the efficacy and safety of UC-MSCs infusion combined with standard IS regimen in pediatric patients with SAA.

There were some noteworthy differences between our study and other works [18, 19, 27] which applied MSCs infusion in AA patients. Firstly, the physical condition and disease background of participants was different. Our study enrolled patients aged between 1 month to 18 years with newly diagnosed SAA, while other studies recruited patients more than 16 years old with refractory or relapsed AA [18, 19, 27]. Secondly, the sources of MSCs were diverse. MSCs applied in other researches [18, 19, 27] were from the BM of healthy donors. The aspiration of BM involved invasive manipulation, and the frequency and differentiation potential of BM-MSCs tapered significantly with certain factors, such as age [28]. Therefore, it is difficult to produce BM-MSCs for utilization on a large scale. By contrast, MSCs in our study were from healthy human umbilical cord tissue. They were separated, screened, passaged and cultured in vitro. To achieve large-scale production of UC-MSCs as “cell medicine”, the “injection of mesenchymal stem cells (umbilical) manufacturing and verification regulation” was also formulated. Moreover, sufficient preclinical studies were conducted using guinea pigs, rabbits, and monkey models [29].

Rosenfeld et al. [30] reported that most of the patients (90%) responded within the first 3 months, with fewer responses occurred between 3 and 6 months or after. A recent multicenter study by Pang et al. [19] showed that the hematologic response occurred almost within 3 months and the overall response rate (ORR) was 28.4%. However, in our study, the ORR at 3 months was only 11% in MSC group, which was lower than that in the controls (22%). When compared with the control group, neither the improvement of blood cell counts, nor the change of T-lymphocytes level reached statistical significance after concomitant use of MSC. It was consistent with the report of Cle et al. [27], but in contrary to the work of Xiao et al. [18] and Pang et al. [19] in which MSC infusion alone can improve the efficacy of AA patients. However, in these two researches, the proportion of nonsevere aplastic anemia (NSAA) patients were 78 and 68%, respectively. Despite of limited number of patients in our study, it provided evidence that UC-MSCs infusion did not necessarily improve the early response rate of childhood SAA. A higher proportion of vSAA patients enrolled (78%) in MSC group, delayed initiation of UC-MSCs infusion (after 2 weeks of rabbit ATG) and the low infusion frequency (a total of three doses) in our study may account for this consequence. Besides, the median ANC at baseline in the group with MSC (0.07 × 10^9^/L) was approximately half of that in control group (0.13 × 10^9^/L), which could result in the lower ORR in MSC users.

The median follow-up period in our study was 48 months, which was longer than other trials [18, 19, 27], thus it enabled us to investigate the long-term efficacy and safety of UC-MSCs utilization in children SAA. The probability of clonal evolution was also a severe adverse event reported in SAA patients, that is, the development of myelodysplastic syndrome (MDS) and acute myelogenous leukemia (AML). Previous study has reported that the incidence of clonal evolution is about 10 to 15% [1]. In our study, there was no clone evolution occurring in both groups at the follow-up endpoint. Moreover, the finding of OS and FFS illustrated that UC-MSCs infusion may not improve the long-term outcomes of pediatric patients with SAA. In MSC group, four (44%) patients suffered treatment failure underwent HSCT during 7-10 months after IST, and we speculated that it may be related to the short time to response and short existence of MSCs in vivo [19]. It was consistent with the studies by Zangi et al. [31] and Ankrum et al. [32] which reported that MSCs cannot persist following infusion and long-term survival of allogeneic MSCs seems to be a major challenge. Therefore, explore new approaches to prolong the persistence of MSCs in vivo may improve the therapeutic effect of this new biological products.

A growing number of laboratory and clinical data have shown that application of MSCs is safe, but there are few articles about the safety of MSCs applied in pediatric patients. In our study, the most notable adverse event developed in MSC group was anaphylactic reaction (rabbit ATG related), however, all the events were mild and can be ameliorated with antiallergic agent. And no UC-MSCs application related death was found in all patients in MSC group. In view of this, UC-MSCs utilization may be well tolerated and safe in childhood SAA.

For some reasons, combination of allogenic UC-MSCs on top of IST was not a conventional treatment for pediatric patients with newly diagnosed SAA in clinical practice. Thus, it was difficult to recruit eligible patients for this study. Allowing for the small sample size of our study and the obvious heterogeneity of enrolled patients, some of the results may not be representative and a prospective study with larger sample size which adopt different doses of UC-MSCs may be necessary.

## Conclusions

This study has preliminarily demonstrated the safety and efficacy of combination of UC-MSCs and standard IST for pediatric patients with newly diagnosed SAA. Concomitant use of IST and UC-MSCs in SAA children is safe but may not necessarily improve the early response rate and long-term outcomes.

## Data Availability

The datasets used and/or analysed during the current study are available from the corresponding author on reasonable request.

## Abbreviations

BM-MSCs: Bone marrow mesenchymal stem cells
AA: Aplastic anemia
UC-MSCs: Umbilical cord mesenchymal stem cells
IST: Immunosuppressive therapy
SAA: Severe aplastic anemia
MSC: Mesenchymal stem cell
ATG: Antithymocyte globulin
PR: Partial response
OS: Overall survival
FFS: Failure-free survival
BM: Bone marrow
PB: Peripheral blood
HSCs: Hematopoietic stem cells
aAA: Acquired aplastic anemia
Tregs: Regulatory T cells
HSCT: Hematopoietic stem cell transplantation
CsA: Cyclosporine
HLA: Human leukocyte antigen
ANC: Absolute neutrophil count
PLT: Platelet
ARC: Absolute reticulocyte count
vSAA: Very severe aplastic anemia
PNH: Paroxysmal nocturnal hemoglobinuria
CR: Complete response
HGB: Hemoglobin
NR: Nonresponse
PerCP: Peridinin chlorophyll
PE: Phycoerythrin
APC: Allophycocyanin
EDTA: Ethylenediaminetetraacetic acid
PBS: Phosphate buffered saline
WBC: White blood cell
aGVHD: Acute graft-versus-host disease
cGVHD: Chronic graft-versus-host disease
HSPCs: Haematopoietic stem/progenitor cells
ORR: Overall response rate
NSAA: Nonsevere aplastic anemia
MDS: Myelodysplastic syndrome
AML: Acute myelogenous leukemia
